# *rac*-1-(2-{[(Benz­yloxy)carbon­yl]amino}­cyclo­hex­yl)pyrrolidin-1-ium chloride

**DOI:** 10.1107/S2414314626006644

**Published:** 2026-06-30

**Authors:** Yoshimi Ichimaru, Masaaki Kurihara

**Affiliations:** a Faculty of Pharmaceutical Sciences Shonan University of Medical Sciences, 16-10 Kamishinano Totsuka-ku Yokohama Kanagawa 244-0806 Japan; Goethe-Universität Frankfurt, Germany

**Keywords:** crystal structure, U50,488H, κ-opioid receptor agonist

## Abstract

The crystal structure of C_19_H_26_N_2_O_2_^+^·Cl^−^, an inter­mediate in the synthesis of the selective κ-opioid receptor agonist U50,488, is reported. The compound crystallizes as a centrosymmetric structure with both (*S*,*S*)- and (*R*,*R*)-enanti­omers in the unit cell. In the crystal, the chloride ion facilitates the formation of enanti­omeric pairs through N—H⋯Cl hydrogen bonds involving both pyrrolidine and the carbamate nitro­gen atoms.

## Abstract 2

The crystal structure of C_19_H_26_N_2_O_2_^+^·Cl^−^, an inter­mediate in the synthesis of the selective κ-opioid receptor agonist U50,488, is reported. The compound crystallizes as a centrosymmetric structure with both (*S*,*S*)- and (*R*,*R*)-enanti­omers in the unit cell. In the crystal, the chloride ion facilitates the formation of enanti­omeric pairs through N—H⋯Cl hydrogen bonds.

## Graphical abstract

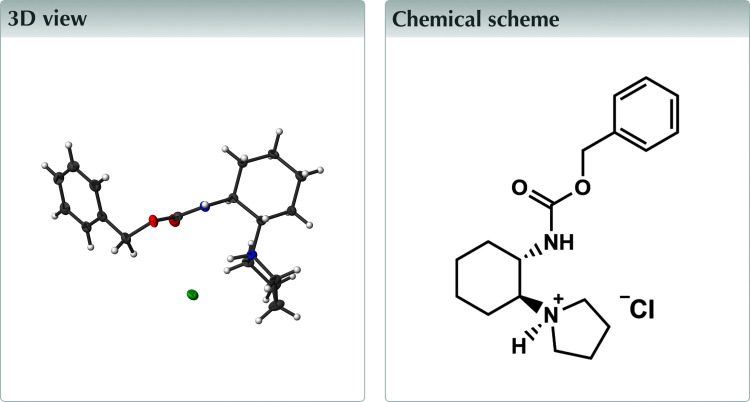

## Structure description

**Figure 1:**
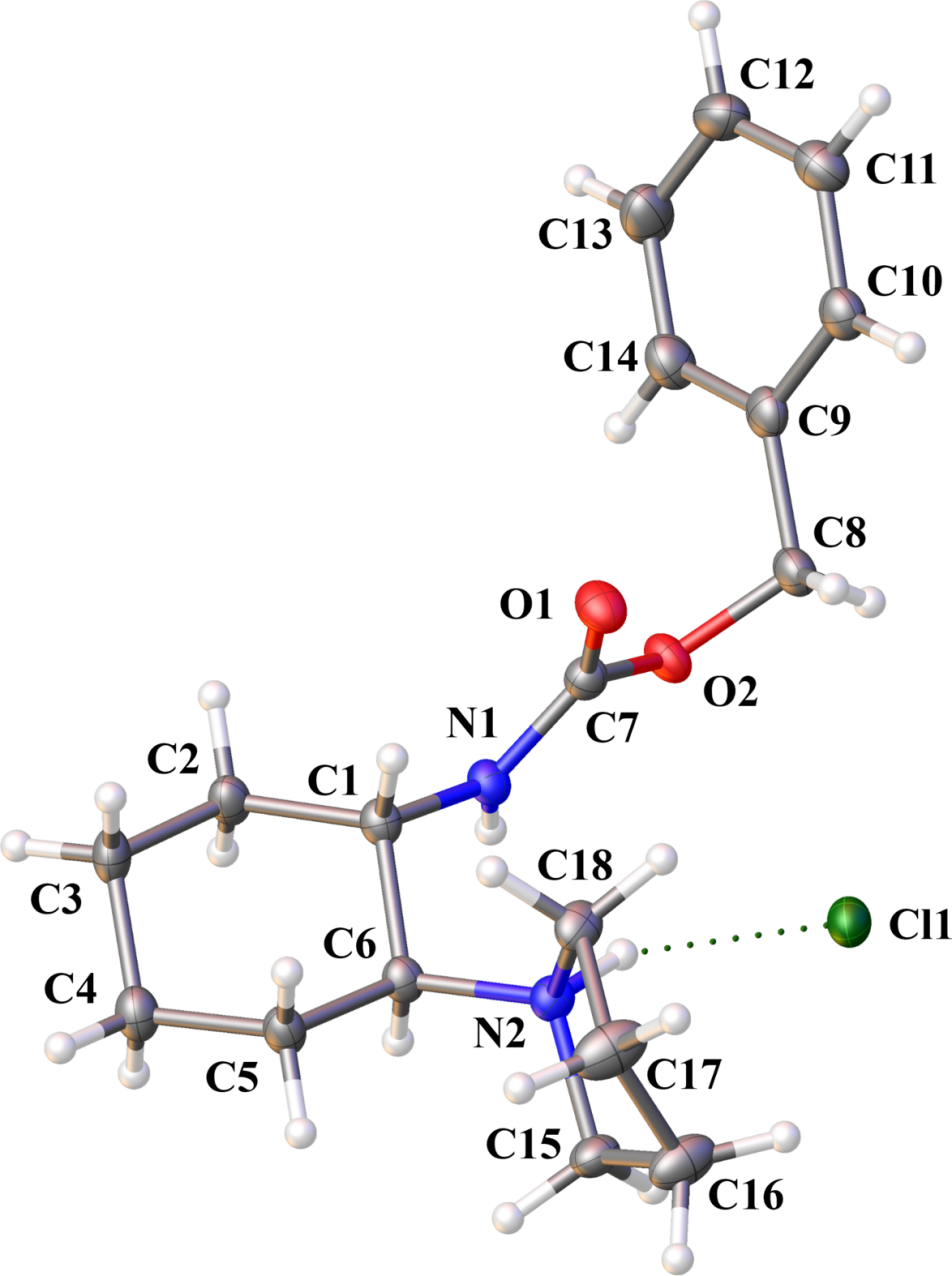
The mol­ecular structure of title compound with displacement ellipsoids drawn at the 50% probability level. The N2—H2⋯Cl1 hydrogen bond is shown as a dashed line.

**Figure 2:**
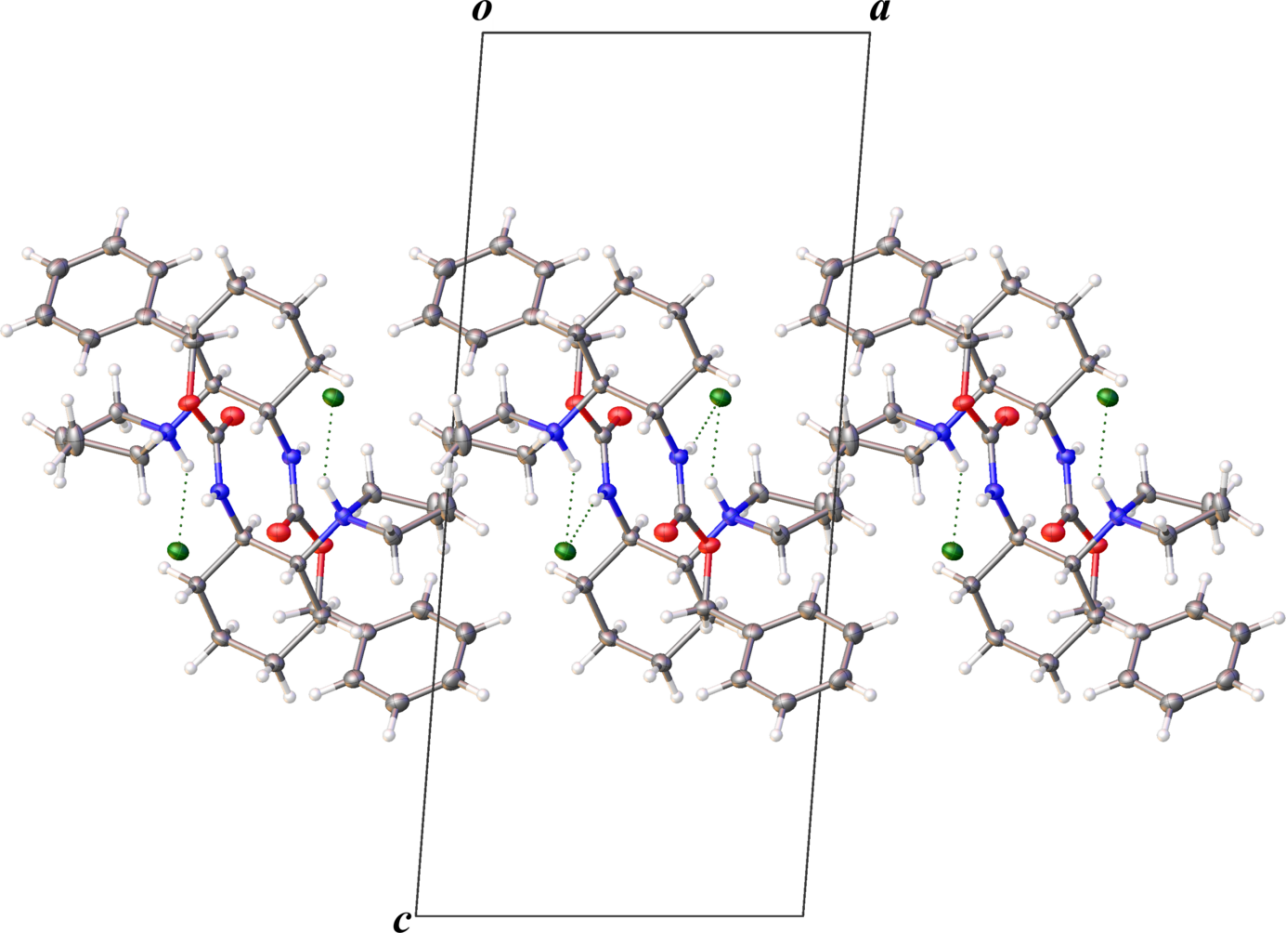
Partial packing diagram viewed along the *b*-axis direction.

The title compound, C_19_H_26_N_2_O_2_^+^·Cl^−^ (Fig. 1 ▸), was synthesized as an inter­mediate in the preparation of U50,488, a selective κ-opioid receptor agonist (Chesis & Welch, 1990 ▸; Kato *et al.*, 2025 ▸). In the arbitrarily chosen asymmetric molecule, atoms C1 and C6 have S configuration but crystal symmetry generates a racemic mixture and the unit cell contains. two mol­ecules of each enanti­omer [(*S*,*S*) and (*R*,*R*)]. Fig. 1 ▸ illustrates the (*S*,*S*)-enanti­omer. The cyclo­hexane ring adopts a chair conformation, with the two substituents in a *trans* configuration. As a result of the significantly reduced basicity of the carbamate-protected nitro­gen atom (benzyl­oxycarbon­yl: Cbz), protonation occurs exclusively at the pyrrolidine nitro­gen atom. The N2—C6 bond length is 1.512 (3) Å, whereas the distance between the Cbz-bonded nitro­gen atom and the cyclo­hexane carbon atom, N1—C1, is slightly shorter at 1.456 (3) Å. The N1—C7 (carbamate carbon­yl) bond is notably shorter at 1.339 (3) Å, reflecting its partial double-bond character. The chloride ion (Cl1) participates in hydrogen bonding with the N2—H2 and N1^i^—H1^i^ groups [symmetry code (i): 1 − *x*, −*y*, 1 − *z*; Table 1 ▸], which facilitates the formation of enanti­omeric pairs. No other significant short-range inter­actions are observed. The crystal packing is illustrated in Fig. 2 ▸.

## Synthesis and crystallization

The title compound was synthesized according to a reported method (Chesis & Welch, 1990 ▸). Single crystals suitable for X-ray analysis were obtained by dissolving the compound in a minimum amount of methanol at 298 K followed by slow evaporation.

## Refinement

Crystal data, data collection and structure refinement details are summarized in Table 2 ▸.

## Supplementary Material

Crystal structure: contains datablock(s) I. DOI: 10.1107/S2414314626006644/bt4201sup1.cif

Structure factors: contains datablock(s) I. DOI: 10.1107/S2414314626006644/bt4201Isup2.hkl

Supporting information file. DOI: 10.1107/S2414314626006644/bt4201Isup3.cml

CCDC reference: 2564391

Additional supporting information:  crystallographic information; 3D view; checkCIF report

## Figures and Tables

**Table 1:** Hydrogen-bond geometry (Å, °)

*D*—H⋯*A*	*D*—H	H⋯*A*	*D*⋯*A*	*D*—H⋯*A*
N2—H2⋯Cl1	1.00	2.20	3.086 (2)	147
N1—H1⋯Cl1^i^	0.88	2.40	3.256 (2)	164

Symmetry code: (i) 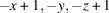.

**Table 2:** Experimental details

Crystal data	
Chemical formula	C_18_H_27_N_2_O_2_^+^·Cl^−^
*M*_r_	338.86
Crystal system, space group	Monoclinic, *P*2_1_/*c*
Temperature (K)	100
*a*, *b*, *c* (Å)	8.8647 (2), 9.7156 (2), 20.2752 (4)
β (°)	94.339 (2)
*V* (Å^3^)	1741.21 (6)
*Z*	4
Radiation type	Cu *K*α
μ (mm^−1^)	2.03
Crystal size (mm)	0.36 × 0.18 × 0.12

Data collection	
Diffractometer	XtaLAB Synergy, Single source at home/near, HyPix3000
Absorption correction	Multi-scan (*CrysAlis PRO*; Rigaku OD, 2025 ▸)
*T*_min_, *T*_max_	0.381, 1.000
No. of measured, independent and observed [*I* > 2σ(*I*)] reflections	14901, 3180, 2676
*R*_int_	0.164
(sin θ/λ)_max_ (Å^−1^)	0.603

Refinement	
*R*[*F*^2^ > 2σ(*F*^2^)], *wR*(*F*^2^), *S*	0.064, 0.178, 1.01
No. of reflections	3180
No. of parameters	208
H-atom treatment	H-atom parameters constrained
Δρ_max_, Δρ_min_ (e Å^−3^)	0.65, −0.41

Computer programs: *CrysAlis PRO* (Rigaku OD, 2025 ▸), *SHELXT* (Sheldrick, 2015*a* ▸), *SHELXL2019/3* (Sheldrick, 2015*b* ▸) and *OLEX2* (Dolomanov *et al.*, 2009 ▸).

## full crystallographic data

### rac-1-(2-{[(Benzyloxy)carbonyl]amino}cyclohexyl)pyrrolidin-1-ium chloride Crystal data

**Table:**

C_18_H_27_N_2_O_2_^+^·Cl^−^	*F*(000) = 728
*M**_r_* = 338.86	*D*_x_ = 1.293 Mg m^−^^3^
Monoclinic, *P*2_1_/*c*	Cu *K*α radiation, λ = 1.54184 Å
*a* = 8.8647 (2) Å	Cell parameters from 8831 reflections
*b* = 9.7156 (2) Å	θ = 4.4–68.1°
*c* = 20.2752 (4) Å	µ = 2.03 mm^−^^1^
β = 94.339 (2)°	*T* = 100 K
*V* = 1741.21 (6) Å^3^	Block, clear colourless
*Z* = 4	0.36 × 0.18 × 0.12 mm

### rac-1-(2-{[(Benzyloxy)carbonyl]amino}cyclohexyl)pyrrolidin-1-ium chloride Data collection

**Table:**

XtaLAB Synergy, Single source at home/near, HyPix3000 diffractometer	3180 independent reflections
Radiation source: micro-focus sealed X-ray tube, PhotonJet-i	2676 reflections with *I* > 2σ(*I*)
Multi-layer mirror optics monochromator	*R*_int_ = 0.164
Detector resolution: 10.0000 pixels mm^-1^	θ_max_ = 68.5°, θ_min_ = 4.4°
ω scans	*h* = −10→8
Absorption correction: multi-scan (CrysAlisPro; Rigaku OD, 2025)	*k* = −11→11
*T*_min_ = 0.381, *T*_max_ = 1.000	*l* = −24→24
14901 measured reflections	

### rac-1-(2-{[(Benzyloxy)carbonyl]amino}cyclohexyl)pyrrolidin-1-ium chloride Refinement

**Table:**

Refinement on *F*^2^	0 restraints
Least-squares matrix: full	Hydrogen site location: inferred from neighbouring sites
*R*[*F*^2^ > 2σ(*F*^2^)] = 0.064	H-atom parameters constrained
*wR*(*F*^2^) = 0.178	*w* = 1/[σ^2^(*F*_o_^2^) + (0.122*P*)^2^] where *P* = (*F*_o_^2^ + 2*F*_c_^2^)/3
*S* = 1.01	(Δ/σ)_max_ = 0.001
3180 reflections	Δρ_max_ = 0.65 e Å^−^^3^
208 parameters	Δρ_min_ = −0.40 e Å^−^^3^

### rac-1-(2-{[(Benzyloxy)carbonyl]amino}cyclohexyl)pyrrolidin-1-ium chloride Special details

**Table:**

Geometry. All esds (except the esd in the dihedral angle between two l.s. planes) are estimated using the full covariance matrix. The cell esds are taken into account individually in the estimation of esds in distances, angles and torsion angles; correlations between esds in cell parameters are only used when they are defined by crystal symmetry. An approximate (isotropic) treatment of cell esds is used for estimating esds involving l.s. planes.

### rac-1-(2-{[(Benzyloxy)carbonyl]amino}cyclohexyl)pyrrolidin-1-ium chloride Fractional atomic coordinates and isotropic or equivalent isotropic displacement parameters (Å^2^)

**Table:**

	*x*	*y*	*z*	*U*_iso_*/*U*_eq_	
Cl1	0.31462 (6)	0.06975 (6)	0.58705 (2)	0.0226 (2)	
O2	0.68572 (18)	0.16204 (17)	0.58084 (7)	0.0215 (4)	
O1	0.57094 (19)	0.37007 (17)	0.56586 (7)	0.0245 (4)	
N1	0.5890 (2)	0.2179 (2)	0.48064 (8)	0.0181 (4)	
H1	0.618740	0.134590	0.470518	0.022*	
N2	0.2644 (2)	0.21635 (19)	0.45218 (9)	0.0182 (4)	
H2	0.322548	0.170743	0.490319	0.022*	
C1	0.5188 (2)	0.3044 (2)	0.42834 (10)	0.0179 (5)	
H1A	0.492998	0.395406	0.447612	0.021*	
C7	0.6101 (2)	0.2600 (2)	0.54351 (10)	0.0183 (5)	
C6	0.3730 (2)	0.2361 (2)	0.39897 (10)	0.0179 (5)	
H6	0.400210	0.142869	0.382696	0.021*	
C2	0.6294 (2)	0.3276 (3)	0.37494 (10)	0.0214 (5)	
H2A	0.717101	0.381232	0.393971	0.026*	
H2B	0.667271	0.237397	0.360558	0.026*	
C10	0.7936 (3)	0.3590 (2)	0.73147 (11)	0.0238 (5)	
H10	0.697356	0.362906	0.749084	0.029*	
C5	0.3017 (2)	0.3171 (3)	0.33981 (10)	0.0211 (5)	
H5A	0.272816	0.410166	0.354293	0.025*	
H5B	0.209221	0.269600	0.321311	0.025*	
C8	0.6864 (3)	0.1819 (3)	0.65116 (10)	0.0229 (5)	
H8A	0.693608	0.091032	0.673296	0.027*	
H8B	0.589551	0.224578	0.661408	0.027*	
C9	0.8150 (3)	0.2712 (2)	0.67860 (11)	0.0221 (5)	
C4	0.4151 (3)	0.3290 (3)	0.28676 (11)	0.0242 (5)	
H4A	0.442449	0.236041	0.271673	0.029*	
H4B	0.368708	0.380359	0.248206	0.029*	
C3	0.5565 (3)	0.4038 (3)	0.31501 (11)	0.0235 (5)	
H3A	0.529334	0.498243	0.328111	0.028*	
H3B	0.629696	0.410856	0.280652	0.028*	
C18	0.1956 (3)	0.3474 (2)	0.47908 (11)	0.0219 (5)	
H18A	0.216769	0.353724	0.527632	0.026*	
H18B	0.236822	0.430222	0.458357	0.026*	
C11	0.9107 (3)	0.4408 (3)	0.75879 (12)	0.0276 (6)	
H11	0.895194	0.499022	0.795300	0.033*	
C15	0.1335 (2)	0.1212 (3)	0.43074 (11)	0.0223 (5)	
H15A	0.114951	0.120563	0.381971	0.027*	
H15B	0.154834	0.025961	0.446143	0.027*	
C14	0.9565 (3)	0.2657 (3)	0.65384 (11)	0.0278 (6)	
H14	0.973179	0.204827	0.618453	0.033*	
C13	1.0742 (3)	0.3482 (3)	0.68013 (12)	0.0342 (6)	
H13	1.170438	0.344158	0.662529	0.041*	
C12	1.0507 (3)	0.4367 (3)	0.73232 (13)	0.0310 (6)	
H12	1.130400	0.494489	0.749859	0.037*	
C16	−0.0008 (3)	0.1796 (3)	0.46268 (15)	0.0321 (6)	
H16A	−0.003924	0.146304	0.508713	0.038*	
H16B	−0.096824	0.154879	0.437297	0.038*	
C17	0.0269 (3)	0.3338 (3)	0.46088 (16)	0.0361 (7)	
H17A	−0.000492	0.371580	0.416221	0.043*	
H17B	−0.032244	0.382295	0.493291	0.043*	

### rac-1-(2-{[(Benzyloxy)carbonyl]amino}cyclohexyl)pyrrolidin-1-ium chloride Atomic displacement parameters (Å^2^)

**Table:**

	*U*^11^	*U*^22^	*U*^33^	*U*^12^	*U*^13^	*U*^23^
Cl1	0.0271 (4)	0.0218 (4)	0.0193 (3)	−0.0014 (2)	0.0049 (2)	0.00011 (19)
O2	0.0269 (9)	0.0217 (9)	0.0154 (7)	0.0035 (6)	−0.0020 (6)	0.0003 (6)
O1	0.0335 (9)	0.0187 (9)	0.0212 (7)	0.0033 (7)	0.0021 (7)	−0.0023 (6)
N1	0.0209 (9)	0.0176 (10)	0.0159 (8)	0.0025 (7)	0.0021 (7)	−0.0010 (7)
N2	0.0182 (9)	0.0171 (10)	0.0196 (9)	0.0015 (7)	0.0038 (7)	−0.0011 (7)
C1	0.0179 (11)	0.0192 (12)	0.0166 (9)	0.0028 (9)	0.0023 (8)	0.0012 (8)
C7	0.0172 (11)	0.0189 (12)	0.0190 (10)	−0.0008 (9)	0.0021 (8)	0.0005 (9)
C6	0.0167 (11)	0.0206 (12)	0.0166 (10)	0.0024 (9)	0.0027 (8)	0.0002 (8)
C2	0.0178 (11)	0.0295 (13)	0.0172 (10)	−0.0003 (9)	0.0026 (8)	0.0016 (9)
C10	0.0253 (11)	0.0265 (14)	0.0192 (10)	0.0020 (10)	0.0002 (9)	0.0031 (9)
C5	0.0187 (11)	0.0279 (13)	0.0167 (10)	0.0000 (9)	0.0010 (8)	0.0022 (9)
C8	0.0282 (12)	0.0245 (13)	0.0156 (10)	−0.0009 (10)	0.0003 (9)	0.0014 (9)
C9	0.0266 (12)	0.0219 (12)	0.0177 (10)	0.0010 (10)	0.0010 (9)	0.0052 (9)
C4	0.0250 (12)	0.0285 (14)	0.0194 (10)	0.0016 (10)	0.0043 (9)	0.0029 (9)
C3	0.0250 (12)	0.0286 (13)	0.0177 (10)	0.0003 (10)	0.0070 (9)	0.0043 (10)
C18	0.0243 (12)	0.0182 (13)	0.0245 (10)	0.0013 (9)	0.0091 (9)	−0.0039 (9)
C11	0.0329 (13)	0.0271 (14)	0.0223 (11)	0.0015 (10)	−0.0018 (10)	−0.0022 (9)
C15	0.0215 (11)	0.0198 (12)	0.0260 (11)	−0.0053 (9)	0.0035 (9)	−0.0026 (9)
C14	0.0301 (13)	0.0323 (14)	0.0214 (10)	0.0013 (11)	0.0040 (9)	−0.0021 (10)
C13	0.0247 (13)	0.0499 (18)	0.0283 (12)	−0.0042 (12)	0.0034 (10)	0.0025 (11)
C12	0.0302 (14)	0.0345 (16)	0.0273 (12)	−0.0087 (11)	−0.0046 (11)	0.0007 (10)
C16	0.0226 (13)	0.0252 (14)	0.0498 (16)	−0.0026 (10)	0.0113 (11)	−0.0074 (11)
C17	0.0224 (13)	0.0257 (14)	0.0610 (18)	0.0014 (10)	0.0092 (12)	−0.0067 (12)

### rac-1-(2-{[(Benzyloxy)carbonyl]amino}cyclohexyl)pyrrolidin-1-ium chloride Geometric parameters (Å, º)

**Table:**

O2—C7	1.361 (3)	C8—C9	1.505 (3)
O2—C8	1.438 (2)	C9—C14	1.387 (3)
O1—C7	1.222 (3)	C4—H4A	0.9900
N1—H1	0.8800	C4—H4B	0.9900
N1—C1	1.456 (3)	C4—C3	1.522 (3)
N1—C7	1.339 (3)	C3—H3A	0.9900
N2—H2	1.0000	C3—H3B	0.9900
N2—C6	1.512 (3)	C18—H18A	0.9900
N2—C18	1.530 (3)	C18—H18B	0.9900
N2—C15	1.521 (3)	C18—C17	1.519 (4)
C1—H1A	1.0000	C11—H11	0.9500
C1—C6	1.532 (3)	C11—C12	1.389 (4)
C1—C2	1.531 (3)	C15—H15A	0.9900
C6—H6	1.0000	C15—H15B	0.9900
C6—C5	1.531 (3)	C15—C16	1.509 (3)
C2—H2A	0.9900	C14—H14	0.9500
C2—H2B	0.9900	C14—C13	1.389 (4)
C2—C3	1.524 (3)	C13—H13	0.9500
C10—H10	0.9500	C13—C12	1.392 (4)
C10—C9	1.394 (3)	C12—H12	0.9500
C10—C11	1.389 (4)	C16—H16A	0.9900
C5—H5A	0.9900	C16—H16B	0.9900
C5—H5B	0.9900	C16—C17	1.520 (4)
C5—C4	1.531 (3)	C17—H17A	0.9900
C8—H8A	0.9900	C17—H17B	0.9900
C8—H8B	0.9900		
			
C7—O2—C8	115.01 (17)	C5—C4—H4A	109.7
C1—N1—H1	118.7	C5—C4—H4B	109.7
C7—N1—H1	118.7	H4A—C4—H4B	108.2
C7—N1—C1	122.57 (19)	C3—C4—C5	109.70 (18)
C6—N2—H2	106.8	C3—C4—H4A	109.7
C6—N2—C18	116.17 (17)	C3—C4—H4B	109.7
C6—N2—C15	112.74 (16)	C2—C3—H3A	109.5
C18—N2—H2	106.8	C2—C3—H3B	109.5
C15—N2—H2	106.8	C4—C3—C2	110.82 (19)
C15—N2—C18	106.98 (16)	C4—C3—H3A	109.5
N1—C1—H1A	108.9	C4—C3—H3B	109.5
N1—C1—C6	109.55 (18)	H3A—C3—H3B	108.1
N1—C1—C2	109.78 (17)	N2—C18—H18A	110.8
C6—C1—H1A	108.9	N2—C18—H18B	110.8
C2—C1—H1A	108.9	H18A—C18—H18B	108.9
C2—C1—C6	110.80 (17)	C17—C18—N2	104.63 (18)
O1—C7—O2	123.21 (19)	C17—C18—H18A	110.8
O1—C7—N1	126.7 (2)	C17—C18—H18B	110.8
N1—C7—O2	110.08 (18)	C10—C11—H11	120.3
N2—C6—C1	109.97 (16)	C10—C11—C12	119.5 (2)
N2—C6—H6	107.6	C12—C11—H11	120.3
N2—C6—C5	112.16 (17)	N2—C15—H15A	110.8
C1—C6—H6	107.6	N2—C15—H15B	110.8
C5—C6—C1	111.58 (18)	H15A—C15—H15B	108.8
C5—C6—H6	107.6	C16—C15—N2	104.98 (18)
C1—C2—H2A	109.1	C16—C15—H15A	110.8
C1—C2—H2B	109.1	C16—C15—H15B	110.8
H2A—C2—H2B	107.9	C9—C14—H14	119.6
C3—C2—C1	112.29 (18)	C9—C14—C13	120.8 (2)
C3—C2—H2A	109.1	C13—C14—H14	119.6
C3—C2—H2B	109.1	C14—C13—H13	120.1
C9—C10—H10	119.5	C14—C13—C12	119.8 (2)
C11—C10—H10	119.5	C12—C13—H13	120.1
C11—C10—C9	121.0 (2)	C11—C12—C13	120.0 (2)
C6—C5—H5A	109.7	C11—C12—H12	120.0
C6—C5—H5B	109.7	C13—C12—H12	120.0
C6—C5—C4	109.61 (18)	C15—C16—H16A	111.1
H5A—C5—H5B	108.2	C15—C16—H16B	111.1
C4—C5—H5A	109.7	C15—C16—C17	103.08 (19)
C4—C5—H5B	109.7	H16A—C16—H16B	109.1
O2—C8—H8A	109.0	C17—C16—H16A	111.1
O2—C8—H8B	109.0	C17—C16—H16B	111.1
O2—C8—C9	112.97 (18)	C18—C17—C16	103.8 (2)
H8A—C8—H8B	107.8	C18—C17—H17A	111.0
C9—C8—H8A	109.0	C18—C17—H17B	111.0
C9—C8—H8B	109.0	C16—C17—H17A	111.0
C10—C9—C8	119.6 (2)	C16—C17—H17B	111.0
C14—C9—C10	118.8 (2)	H17A—C17—H17B	109.0
C14—C9—C8	121.6 (2)		
			
O2—C8—C9—C10	145.6 (2)	C2—C1—C6—C5	−53.9 (2)
O2—C8—C9—C14	−36.2 (3)	C10—C9—C14—C13	−1.4 (4)
N1—C1—C6—N2	59.7 (2)	C10—C11—C12—C13	−1.9 (4)
N1—C1—C6—C5	−175.20 (16)	C5—C4—C3—C2	58.8 (3)
N1—C1—C2—C3	173.48 (19)	C8—O2—C7—O1	13.8 (3)
N2—C6—C5—C4	−177.93 (19)	C8—O2—C7—N1	−167.13 (18)
N2—C18—C17—C16	31.3 (3)	C8—C9—C14—C13	−179.5 (2)
N2—C15—C16—C17	34.8 (3)	C9—C10—C11—C12	1.1 (4)
C1—N1—C7—O2	−176.42 (17)	C9—C14—C13—C12	0.5 (4)
C1—N1—C7—O1	2.6 (3)	C18—N2—C6—C1	69.0 (2)
C1—C6—C5—C4	58.2 (2)	C18—N2—C6—C5	−55.8 (2)
C1—C2—C3—C4	−55.3 (3)	C18—N2—C15—C16	−15.5 (2)
C7—O2—C8—C9	−87.6 (2)	C11—C10—C9—C8	178.7 (2)
C7—N1—C1—C6	−118.2 (2)	C11—C10—C9—C14	0.6 (3)
C7—N1—C1—C2	119.9 (2)	C15—N2—C6—C1	−167.05 (18)
C6—N2—C18—C17	117.1 (2)	C15—N2—C6—C5	68.2 (2)
C6—N2—C15—C16	−144.4 (2)	C15—N2—C18—C17	−9.8 (2)
C6—C1—C2—C3	52.3 (3)	C15—C16—C17—C18	−41.2 (3)
C6—C5—C4—C3	−60.1 (3)	C14—C13—C12—C11	1.2 (4)
C2—C1—C6—N2	−179.06 (18)		

### rac-1-(2-{[(Benzyloxy)carbonyl]amino}cyclohexyl)pyrrolidin-1-ium chloride Hydrogen-bond geometry (Å, º)

**Table:**

*D*—H···*A*	*D*—H	H···*A*	*D*···*A*	*D*—H···*A*
N2—H2···Cl1	1.00	2.20	3.086 (2)	147
N1—H1···Cl1^i^	0.88	2.40	3.256 (2)	164

Symmetry code: (i) −*x*+1, −*y*, −*z*+1.
